# Continuous Catheter Techniques Versus Single-Injection Nerve Blocks: A Comprehensive Review of Postoperative Pain Management Strategies

**DOI:** 10.7759/cureus.70040

**Published:** 2024-09-23

**Authors:** Akansha Singhal, Karuna Taksande

**Affiliations:** 1 Anaesthesiology, Jawaharlal Nehru Medical College, Datta Meghe Institute of Higher Education and Research, Wardha, IND

**Keywords:** continuous catheter techniques, efficacy, patient outcomes, postoperative pain management, regional anesthesia, single-injection nerve blocks

## Abstract

Effective postoperative pain management is crucial for optimizing patient recovery and enhancing surgical outcomes. This review compares two prominent regional anesthesia techniques, continuous catheter techniques and single-injection nerve blocks, focusing on their efficacy, safety, and impact on patient outcomes. Single-injection nerve blocks involve administering a one-time anesthetic dose to a specific nerve or plexus, offering immediate but transient pain relief. In contrast, continuous catheter techniques utilize a catheter to deliver a continuous infusion of anesthetic, providing extended analgesia. The review synthesizes current evidence on the effectiveness of each method, highlighting that single-injection blocks are advantageous for their simplicity and rapid onset but may require supplementary pain management for longer procedures. Continuous catheter techniques, while offering prolonged pain relief, carry risks such as infection and catheter displacement. The comparative analysis of these techniques reveals that both have distinct roles in postoperative care, with choice depending on the surgical procedure and patient needs. Patient satisfaction, recovery times, and overall outcomes are critical factors in determining the optimal pain management strategy. Future research should focus on refining these techniques and exploring innovations to improve patient care and pain management outcomes. This review provides insights for clinicians to make informed decisions about postoperative pain management to enhance patient comfort and recovery.

## Introduction and background

Effective postoperative pain management is critical for optimizing patient recovery and improving overall surgical outcomes [1]. Persistent pain following surgery can lead to increased morbidity, extended hospital stays, and delayed functional recovery. Proper management of pain not only alleviates discomfort but also helps prevent complications associated with inadequate pain control, such as chronic pain syndromes and impaired mobility [2]. By addressing pain effectively, healthcare providers can significantly enhance patient comfort and facilitate a smoother recovery process [3].

In clinical practice, various pain management strategies are utilized to address postoperative pain [4]. These strategies range from pharmacological approaches, including opioids and nonsteroidal anti-inflammatory drugs (NSAIDs), to regional anesthesia techniques such as nerve blocks. Among the regional techniques, single-injection nerve blocks and continuous catheter techniques are two prominent methods. Single-injection nerve blocks involve administering a single anesthetic dose to a specific nerve or nerve plexus, providing effective but temporary pain relief [3]. On the other hand, continuous catheter techniques involve placing a catheter near a nerve or within the epidural space to deliver a continuous infusion of anesthetic agents, which offers prolonged analgesia [5].

This review aims to systematically compare continuous catheter techniques with single-injection nerve blocks in the context of postoperative pain management. The comparison focuses on evaluating several key aspects: the efficacy of each technique in providing pain relief and its impact on patient comfort and functional recovery; the safety profiles, including the incidence of complications and adverse effects; and overall patient outcomes, such as satisfaction, recovery times, and long-term pain management. By examining and synthesizing the available evidence, this review seeks to provide valuable insights into the benefits and limitations of these pain management strategies, thereby aiding clinical decision-making and enhancing postoperative care.

## Review

Pain management strategies

Pain management strategies in postoperative care often incorporate single-injection nerve blocks and continuous catheter techniques, offering distinct benefits and specific applications [6]. Single-injection nerve blocks involve administering a single dose of local anesthetic near a specific nerve or nerve plexus, providing targeted analgesia for a limited duration. This technique is widely used in various surgical procedures to manage postoperative pain effectively [7]. Common types of single-injection nerve blocks include peripheral nerve blocks and neuraxial blocks. Peripheral nerve blocks target specific nerves to block sensation in designated areas; for example, the interscalene block is frequently used for shoulder surgeries, while the femoral nerve block is commonly employed in knee surgeries [7]. Neuraxial blocks involve injecting anesthetic into the epidural or intrathecal space, including epidural blocks for larger areas, often utilized during labor or major abdominal surgeries, and spinal blocks, which provide rapid-onset anesthesia for lower body surgeries [8]. In contrast, continuous catheter techniques involve the placement of a catheter near a nerve or nerve plexus to allow for the continuous infusion of local anesthetic, thereby providing prolonged analgesia. This method is particularly advantageous for managing postoperative pain over extended periods [9]. Common types of continuous catheter techniques include continuous peripheral nerve blocks and epidural analgesia. Continuous peripheral nerve blocks involve placing a catheter adjacent to a peripheral nerve, enabling the ongoing delivery of anesthetic for various surgeries, including orthopedic and abdominal procedures. Epidural analgesia, on the other hand, involves placing a catheter in the epidural space to continuously deliver local anesthetics and/or opioids, making it a frequent choice for labor analgesia and postoperative pain management in abdominal and thoracic surgeries [10]. Single-injection nerve blocks and continuous catheter techniques are essential components of multimodal pain management, allowing healthcare providers to tailor approaches based on the specific type of surgery, anticipated pain levels, and individual patient factors [11]. A comparison of continuous catheter techniques versus single-injection nerve blocks in postoperative pain management is provided in Table 1.

**Table 1:** Comparison of continuous catheter techniques versus single-injection nerve blocks in postoperative pain management

Aspect	Continuous catheter techniques	Single-injection nerve blocks
Duration of pain relief [12]	Extended; can last several days with continuous infusion.	Shorter duration, typically lasting a few hours to a single day.
Ease of use [12]	Requires more technical skill and equipment for insertion.	Easier to perform with less technical skill required.
Complication rate [12]	Higher risk of infection, catheter displacement, or leakage.	Lower risk of complications compared to continuous techniques.
Patient satisfaction [12]	Generally higher due to sustained pain relief over time.	It may vary; satisfaction decreases as the block wears off.
Control over pain management [12]	Allows titration of medication dosage based on patient needs.	Fixed dosage; no ability to adjust post-procedure.
Cost [12]	Typically, it is higher due to equipment and extended monitoring needs.	Lower upfront cost with fewer resources required.
Postoperative recovery [12]	It may facilitate quicker mobilization due to prolonged pain control.	Faster initial recovery, but pain may return earlier.
Side effects [12]	Potential for continuous numbness and motor weakness in the area.	Less likelihood of prolonged numbness or motor dysfunction.
Suitability for complex surgeries [12]	Preferred for longer surgeries and cases with expected severe pain.	Suitable for short, less invasive procedures with mild pain.
Clinical settings [12]	Often used in hospitals or specialized clinics with monitoring.	It can be used in outpatient or day surgery settings.

Efficacy of pain relief

Single-Injection Nerve Blocks

Single-injection nerve blocks are a widely favored option for postoperative pain management due to their simplicity and effectiveness. These blocks typically provide analgesia for 3-18 hours, influenced by factors such as the specific local anesthetic used, the technique employed, and individual patient responses [13]. While single-injection nerve blocks can effectively reduce pain immediately following surgery, their relatively short duration may not be sufficient for patients undergoing more extensive or painful procedures. As the anesthetic effects wear off, patients may experience a resurgence of pain, often leading to an increased reliance on systemic opioids for pain control [3]. In terms of effectiveness, single-injection nerve blocks have been shown to significantly lower postoperative pain scores and reduce the need for opioid analgesics [14]. However, the degree of pain relief can vary widely among patients, and some may find the analgesic effect insufficient for managing their pain adequately. Studies suggest that while these blocks can improve pain management in the immediate postoperative period, they may not always provide clinically significant pain relief for all patients, particularly those with higher pain expectations or those undergoing more extensive surgical interventions [14]. Patient satisfaction with single-injection nerve blocks is generally high, especially when patients experience meaningful pain relief. However, the impact on functional outcomes can be mixed [15]. While many patients report reduced pain levels, some studies indicate that overall recovery and functional performance may not differ significantly from those receiving traditional analgesic approaches. This variability highlights the importance of tailoring pain management strategies to the individual needs of patients and the specific surgical context [15].

Continuous Catheter Techniques

In contrast, continuous catheter techniques, such as peripheral nerve blocks, provide a more prolonged and sustained approach to pain relief [16]. These techniques can offer analgesia for 2-3 days, making them particularly advantageous for managing postoperative pain beyond the short-term relief of single-injection blocks. The extended duration of analgesia can significantly enhance the overall postoperative experience, enabling patients to engage more actively in rehabilitation and recovery without the burden of uncontrolled pain [16]. The effectiveness of continuous catheter techniques in pain relief is well-documented. Research shows that these methods are associated with lower pain scores and reduced opioid consumption over an extended postoperative period [17]. This sustained pain control not only enhances patient comfort but also reduces the potential side effects and complications of opioid use, such as nausea, constipation, and respiratory depression. Consequently, continuous catheter techniques are often considered a superior option for patients undergoing major surgeries or those anticipated to experience higher pain levels [17]. Patient satisfaction tends to be higher among those receiving continuous catheter techniques, largely due to the extended pain relief and decreased need for additional analgesics [18]. Moreover, these techniques can lead to improved functional outcomes, as patients are often better able to participate in physical therapy and other recovery activities without significant pain. However, it is important to consider the complexities of managing a catheter, including proper placement, maintenance, and monitoring for potential complications such as infection or catheter dislodgment. Patient education and adherence to the pain management regimen are crucial for achieving optimal results [18].

Safety and complications

Single-injection nerve blocks and continuous catheter techniques are generally safe procedures but carry inherent risks. Understanding these potential complications and implementing prevention strategies is essential for optimizing patient outcomes [19]. Common complications associated with single-injection nerve blocks include local anesthetic systemic toxicity (LAST), nerve damage, infection, and hematoma formation. LAST occurs when local anesthetic inadvertently enters the bloodstream, potentially leading to serious cardiovascular and neurological complications [20]. Nerve damage can result from direct trauma during the injection, manifesting as weakness, numbness, or pain in the affected area. While relatively uncommon, infection at the injection site remains a risk. Hematoma formation at the injection site can also occur, potentially compressing surrounding structures and leading to additional complications [21]. Certain patient-specific factors, such as obesity, diabetes, or pre-existing neuropathy, may increase the risk of complications. Technical factors, including the skill of the practitioner and the use of ultrasound guidance, can also significantly influence outcomes [22]. Ultrasound guidance can improve accuracy, reducing the risk of nerve injury and hematoma. Proper patient selection and thorough pre-procedural assessments are vital for identifying those at higher risk for complications. Post-procedure monitoring for signs of LAST or neurological deficits is crucial for early intervention [22]. Continuous catheter techniques, while offering extended analgesia, come with their own set of complications. Infection is a primary concern, particularly if the catheter remains in place for extended periods [23]. Catheter dislodgement can lead to inadequate pain control, potentially necessitating re-insertion. As with single-injection blocks, there is a risk of nerve damage, especially if the catheter is improperly placed. Intra-articular infusion of local anesthetics can result in hydrolysis, which damages cartilage, particularly in joints like the shoulder [23]. Prolonged catheter placement increases the risk of both infection and dislodgement. Patient factors, such as movement or activity levels post-surgery, can impact catheter stability [24]. Adhering to strict aseptic techniques during catheter insertion and maintenance can minimize infection risks. Educating patients on catheter care and activity restrictions can help prevent dislodgement. Regular monitoring and assessing the catheter site for signs of infection or complications are essential for timely intervention [24]. A comparison of the safety and complications associated with continuous catheter techniques versus single-injection nerve blocks is presented in Table 2.

**Table 2:** Safety and complications of continuous catheter techniques versus single-injection nerve blocks

Aspect	Continuous catheter techniques	Single-injection nerve blocks
Infection risk [25]	Higher risk due to prolonged catheter presence.	Lower risk as it is a single injection with no foreign material left in the body.
Catheter displacement [26]	Risk of catheter displacement, requiring repositioning or reinsertion.	It is not applicable, as no catheter is involved.
Local anesthetic toxicity [27]	Increased risk if infusion is not properly monitored.	Lower risk due to a single, controlled dose.
Nerve damage [28]	Rare but prolonged catheter presence could irritate nerves.	Rare, but possible due to direct injection into or near the nerve.
Motor function impairment [29]	Potential for prolonged motor weakness due to continuous delivery of anesthetic.	Motor weakness resolves once the block wears off.
Hematoma [30]	Rare, but possible in patients with coagulation issues.	Rare, with lower risk compared to continuous catheter techniques.
Respiratory depression [31]	Possible if anesthetic spreads to unintended areas (e.g., epidural space).	Uncommon with correct technique and dose.
Allergic reactions [32]	Rare, but possible with prolonged exposure to anesthetic agents.	Rare, short-term exposure reduces the likelihood.
Post-procedural monitoring [33]	Requires close monitoring for complications, particularly infections.	Minimal monitoring is required after the procedure.
Patient mobility [34]	It may limit mobility due to the attached equipment.	Allows for easier mobilization once the block wears off.

Comparative analysis

Recent research has compared continuous peripheral nerve catheters with single-injection nerve blocks for postoperative pain management, yielding important insights into their relative effectiveness. A systematic review and meta-analysis concluded that continuous peripheral nerve blocks offer superior pain control, reduced opioid consumption, and less postoperative nausea compared to single-injection blocks [11]. Another review highlighted that while continuous catheter techniques provide more prolonged and effective analgesia, they require greater expertise and are associated with higher risks. In contrast, single-injection blocks are simpler and safer but offer a shorter duration of pain relief [23]. A meta-analysis of randomized trials comparing continuous and single-shot adductor canal blocks for total knee arthroplasty found minimal differences in analgesic outcomes between the two techniques. The authors suggested that combining a multimodal pain regimen with a single-shot block can offer pain control comparable to that of a continuous catheter [35]. However, one study noted an increased risk of injection site complications and minor adverse events with continuous interscalene blocks compared to single-injection blocks following rotator cuff repair. Overall, the evidence indicates that continuous catheters provide better pain relief and carry higher complication rates. In contrast, single-shot blocks are simpler but may not offer adequate analgesia beyond the first postoperative day [35]. The cost-effectiveness of these two techniques varies based on several factors. Continuous catheters involve more expertise for placement and maintenance, leading to higher procedural costs. However, the superior analgesia provided by continuous catheters may result in reduced overall costs, such as decreased opioid use, shorter hospital stays, and fewer unplanned healthcare interventions [23]. One study identified unexpected costs associated with interscalene nerve catheters for shoulder surgery, emphasizing the need for thorough cost analysis [36]. Continuous blocks may be more cost-effective for surgeries expected to cause severe pain that extends beyond the duration of single-injection blocks. In conclusion, while continuous catheters have higher initial procedural costs, their enhanced analgesic benefits may offset other healthcare expenses in certain situations. Nonetheless, further research is necessary to determine the cost-effectiveness of the two techniques [36].

Patient-centered considerations

The choice of pain management technique can significantly influence a patient's overall experience and satisfaction with their care. Patients often have varying preferences based on their unique situations, shaped by multiple factors. Anticipated pain levels are crucial; for instance, patients undergoing more invasive procedures may prefer continuous catheter techniques due to their extended analgesic effects. Conversely, those expecting milder pain might lean toward single-injection nerve blocks for their simplicity and lower risk profile [37]. Mobility is another key consideration. Patients who prioritize early mobilization and active participation in rehabilitation may favor techniques that provide effective pain control without restricting movement. Personal experiences, previous encounters with pain management strategies, and concerns about potential risks and complications also significantly impact patient preferences. Engaging patients in decision-making ensures they address their individual needs and goals, leading to a more personalized and satisfactory pain management approach [38]. The effectiveness of the chosen pain management technique profoundly affects a patient's postoperative recovery and rehabilitation journey. Effective pain control facilitates early mobilization, preventing complications such as deep vein thrombosis and promoting quicker recovery. Continuous catheter techniques often provide superior analgesia, enabling patients to manage pain more effectively and participate actively in physical therapy [3]. This can lead to faster recovery times and a smoother transition to daily activities. However, while single-injection nerve blocks may offer adequate pain relief for shorter procedures, their limited duration might necessitate additional interventions for pain management, potentially delaying recovery [39]. The overall impact on recovery is multifaceted, depending on other factors such as the specific surgical procedure, patient comorbidities, and the quality of postoperative care. Therefore, a comprehensive approach that considers pain management techniques and individual patient factors is essential for optimizing recovery outcomes [39]. A summary of patient-centered considerations for continuous catheter techniques versus single-injection nerve blocks is provided in Table 3.

**Table 3:** Patient-centered considerations for continuous catheter techniques versus single-injection nerve blocks

Aspect	Continuous catheter techniques	Single-injection nerve blocks
Patient comfort [40]	It may cause discomfort due to the presence of the catheter over several days.	Generally, it is more comfortable as it involves a single injection.
Pain control duration [3]	Provides long-lasting pain control, reducing the need for additional medications.	Shorter duration of pain control; may require additional interventions or medications as the block wears off.
Mobility [41]	Limited mobility due to the catheter and associated equipment.	Greater mobility as there is no external device after the injection.
Independence post-surgery [42]	Requires continuous monitoring or assistance for catheter care.	Patients can often resume daily activities after the block wears off.
Impact on daily activities [43]	Potential disruption in normal activities due to the catheter and attached equipment.	Minimal impact on daily activities once the block has worn off.
Patient anxiety [40]	It may cause anxiety due to the need for catheter management and concerns over potential complications.	Generally, it's less anxiety-inducing as it's a single, short-term procedure.
Cost to patient [44]	Higher cost due to extended pain control and more frequent monitoring.	Lower cost, as it involves a single procedure with minimal follow-up.
Suitability for outpatient surgery [45]	Less suitable for outpatient settings due to the need for prolonged monitoring.	Ideal for outpatient procedures due to the short duration of the block.
Patient satisfaction [46]	Higher satisfaction for patients needing prolonged pain control, especially after major surgeries.	High satisfaction for patients undergoing minor surgeries or procedures.
Follow-up care [47]	Requires follow-up for catheter removal and monitoring for complications.	Minimal follow-up care is required once the block has worn off.

Future directions

Emerging technologies and advancements in pain management are significantly reshaping treatment options, offering new possibilities for enhancing patient care. One of the most promising areas is neuromodulation, which involves altering nerve activity through the targeted delivery of stimuli, such as electrical impulses [48]. Techniques like spinal cord stimulation (SCS) and peripheral nerve stimulation (PNS) have shown substantial promise in managing chronic pain and reducing reliance on opioids. Additionally, the rise of telemedicine has greatly improved access to pain management services, enabling remote monitoring and follow-up care. Automated mobile applications further enhance this by facilitating symptom tracking and improving communication between patients and providers, ultimately leading to better pain management outcomes [49]. Another exciting development is using virtual reality (VR) and augmented reality (AR) technologies. These immersive tools are being utilized to distract patients from pain during procedures and rehabilitation, providing a non-pharmacological approach to pain relief. In the pharmaceutical domain, advancements in biotechnology are leading to the development of new drugs that offer improved efficacy and reduced side effects [50]. Innovative techniques such as magnetoreception are being explored to deliver medications more effectively to targeted areas. Furthermore, regenerative medicine approaches, including stem cell therapy and platelet-rich plasma (PRP) injections, aim to harness the body's natural healing processes to address the underlying causes of pain, particularly in musculoskeletal conditions [51]. Artificial intelligence (AI) is also making significant strides in pain management. By integrating AI and machine learning into pain management protocols, healthcare providers can analyze vast amounts of data to optimize treatment plans, potentially improving patient outcomes through personalized interventions [52].

Despite these advancements, significant knowledge gaps remain, warranting further investigation. One critical area is the long-term efficacy and safety of emerging technologies. More research is needed to evaluate the sustained benefits and potential adverse effects of techniques such as neuromodulation and regenerative therapies [53]. Additionally, there is a pressing need to personalize pain management strategies. Studies should focus on how genetic and biomolecular factors influence pain perception and treatment responses, which could lead to more tailored approaches that improve efficacy while minimizing side effects [53]. Research should also explore integrating different technologies to enhance pain management strategies. For instance, combining telemedicine with wearable devices could provide real-time monitoring capabilities and enable timely interventions. Furthermore, understanding the impact of socioeconomic factors on access to pain management technologies and outcomes is crucial. Investigating this area can inform policies to reduce disparities in access to effective pain management [54]. Lastly, a deeper understanding of the psychosocial aspects of pain and their interaction with technological interventions is essential. Research in this domain could lead to more holistic treatment strategies that address pain's physical and emotional components. In conclusion, the future of pain management lies in the convergence of innovative technologies and personalized care approaches. Ongoing research will be vital in fully realizing the potential of these advancements and addressing existing gaps in knowledge [55]. The future directions for continuous catheter techniques and single-injection nerve blocks in pain management are summarized in Table 4.

**Table 4:** Future directions for continuous catheter techniques and single-injection nerve blocks in pain management AI: artificial intelligence

Aspect	Continuous catheter techniques	Single-injection nerve blocks
Technological advancements [56]	Development of wireless or portable catheter systems for greater mobility and convenience.	Improved precision in drug delivery and nerve targeting.
Integration with pain management software [57]	Integration with AI-based pain management software for real-time adjustments in dosage.	AI-assisted techniques to optimize block placement and dosage based on patient anatomy.
Minimally invasive techniques [58]	Refinement in catheter insertion techniques to reduce invasiveness and patient discomfort.	Enhanced needle design for even less invasive procedures.
Customization of pain relief [59]	Personalized infusion protocols based on patient genetics and pain sensitivity.	Single injections are tailored to specific patient needs and procedure types.
Long-term pain management solutions [9]	Exploration of combination therapies using catheters for chronic pain conditions.	Potential for longer-acting anesthetics that extend block duration.
Reduction in complication rates [60]	Research into antimicrobial-coated catheters to reduce infection risks.	Continued advancements to reduce rare nerve injury or toxicity risks.
Improved patient monitoring [61]	Development of smart catheters that can monitor and transmit data on nerve health and anesthetic levels.	Enhanced real-time monitoring for better post-procedural care.
Cost-effectiveness [62]	Efforts to reduce the cost of continuous catheter systems through new materials and production techniques.	Further cost reduction through simplified and more efficient procedures.
Patient education and autonomy [63]	Increasing patient autonomy by developing self-managing catheter systems with remote assistance.	Improved patient education on single-injection safety and potential side effects.
Expanded use cases [64]	Investigating broader applications in non-surgical, long-term pain conditions (e.g., cancer pain management).	Potential expansion into new fields such as emergency or field medicine.

## Conclusions

Both continuous catheter techniques and single-injection nerve blocks offer valuable approaches to postoperative pain management, each with distinct advantages and limitations. Single-injection nerve blocks provide effective, immediate pain relief with a straightforward procedure, making them suitable for shorter or less complex surgeries. However, their limited duration of action may necessitate additional pain management strategies for extended postoperative periods. Conversely, continuous catheter techniques offer prolonged analgesia through ongoing anesthetic infusion, which can significantly enhance pain control and patient comfort for more extended recovery periods. Despite the benefits of continuous techniques, they are associated with potential complications, such as infection or catheter dislodgement, and may involve more complex procedures. Evaluating these techniques' relative efficacy, safety, and patient outcomes is essential for optimizing postoperative pain management. Future research should continue to explore innovations in both approaches to further refine their use and improve patient care. Ultimately, selecting the most appropriate pain management strategy requires careful consideration of the individual patient's needs, the type of surgery, and the overall clinical context.
